# Online and Offline Recruitment of Young Women for a Longitudinal Health Survey: Findings From the Australian Longitudinal Study on Women’s Health 1989-95 Cohort

**DOI:** 10.2196/jmir.4261

**Published:** 2015-05-04

**Authors:** Deborah Loxton, Jennifer Powers, Amy E Anderson, Natalie Townsend, Melissa L Harris, Ryan Tuckerman, Stephanie Pease, Gita Mishra, Julie Byles

**Affiliations:** ^1^Priority Research Centre for Gender, Health and AgeingSchool of Medicine and Public HealthUniversity of NewcastleCallaghanAustralia; ^2^Centre for Longitudinal and Life Course ResearchSchool of Population HealthUniversity of QueenslandHerstonAustralia

**Keywords:** cohort studies, longitudinal studies, social media, women’s health

## Abstract

**Background:**

In 2012, we set out to recruit a cohort of at least 10,000 women aged 18-23 from across Australia. With recent research demonstrating the inadequacy of traditional approaches to recruiting women in this age group, we elected to conduct open recruiting.

**Objective:**

Our aim was to report on the overall success of open recruiting and to evaluate the relative success of a variety of recruitment methods in terms of numbers and demographics.

**Methods:**

We used referrals, Facebook, formal advertising, and incentives in order to recruit the cohort.

**Results:**

In all, 17,069 women were recruited for the longitudinal online survey, from 54,685 initiated surveys. Of these women, most (69.94%, n=11,799) who joined the longitudinal cohort were recruited via Facebook, 12.72% (n=2145) via the fashion promotion, 7.02% (n=1184) by referral, 4.9% (n=831) via other Web activities, and 5.4% (n=910) via traditional media.

**Conclusions:**

Facebook was by far the most successful strategy, enrolling a cohort of women with a similar profile to the population of Australian women in terms of age, area of residence, and relationship status. Women recruited via fashion promotion were the least representative. All strategies underrepresented less educated women—a finding that is consistent with more traditional means of recruiting. In conclusion, flexibility in recruitment design, embracing new and traditional media, adopting a dynamic responsive approach, and monitoring the results of recruiting in terms of sample composition and number recruited led to the successful establishment of a new cohort.

## Introduction

Recruiting participants has become increasingly challenging in the face of telemarketing, mass direct marketing, privacy concerns, and expectations of rewards for survey completion [1]. Traditional methods of recruiting participants, such as mailed invitations to random samples drawn from existing databases [2-4] have become less effective and more expensive [5]. Furthermore, changes to privacy laws have resulted in limited access to some national databases, even for the purposes of health research [6]. These findings point to the need for new methods that are tailored to recruit young people in the present day. This paper reports on the methods that were developed to recruit a new national cohort of women aged 18-23 into an existing longitudinal study on women’s health.

Advances in technology offer opportunities for adapting recruitment methods to reach younger generations. Online recruitment has been shown to be more cost-effective than postal recruitment for young people [5,7,8]. Furthermore, social media, particularly Facebook [9], has been identified by young women as being a trusted communication channel that could entice them to participate in research [10]. Importantly, advertising on Facebook has been found to be an effective recruitment tool, with broad populations of young adults successfully recruited in Australia, Canada, and United States [8,11-13]. Additionally, there is some evidence that samples recruited through Facebook are ethnically diverse and geographically representative [8,11,12]. However, there are mixed results regarding whether Facebook advertising can be used to successfully recruit a sample that is demographically representative of the target population, particularly with regard to age, education level, and income [8,12,14]. In addition, not all young women use social media, and those who do not engage with social networking sites have been found to be less economically stable and less educated than those who participate in social media [15]. Therefore, recruiting entirely through social media may result in a biased sample.

There is some evidence that using a number of recruitment methods is an effective strategy. In the United States, smokers were successfully recruited via traditional and online methods including media, word of mouth, email referrals, medical Internet media, Google AdWords [16], and social networking sites [17]. Similarly, Ramo et al [18] successfully recruited young American adults into an online tobacco use survey through a variety of methods, including online classified advertisements, paid advertising through an Internet marketing company, and purchased completed surveys through an online survey sampling company. When specifically focused on young women, Harris et al [19] successfully recruited women aged 18-23 years into a study of contraception use using Facebook advertising supported by a social media presence, in conjunction with network snowballing, poster distribution, and attending tertiary education events to raise awareness of the study. Despite these findings, the relative success of each type of recruiting method in terms of demographic representativeness has not been examined.

The aim of this study was to recruit a cohort of at least 10,000 women aged 18-23 years into the Australian Longitudinal Study on Women’s Health (ALSWH). This paper reports on the open recruiting campaign and the variety of methods that were used to recruit the cohort. The relative success of the different recruiting strategies in terms of the number of women recruited and the demographic representativeness of the subsamples that resulted from each strategy are reported.

## Methods

### Study Overview

The ALSWH is a national study that recruited over 40,000 women into three age cohorts in 1996 [20]. The original three cohorts, born 1973-78, 1946-51, and 1921-26, were randomly sampled from the Medicare (national health insurance) database and have completed mailed omnibus health surveys every 3 years [4,21,22]. Random sampling from the Medicare database for the new cohort was not feasible due to the very low response rate (6%) reported by another study that attempted to recruit women aged 18-23 years using this method [5]. The ALSWH aims to assess the health and well-being of Australian women to provide an evidence base for both policy and practice. The study measures multiple aspects of health including physical health, mental health, health service use, as well as the social and environmental determinants of health. In 2011, ALSWH was funded to incorporate the use of online surveys as well as to recruit a new cohort of women aged 18-23 years who would complete annual online health surveys.

### Participants

Eligibility criteria for the 1989-95 cohort included living in Australia, being a female aged 18-23, possessing a Medicare (ie, Australian national health insurer) number (Australian and New Zealand citizens and permanent residents living in Australia are eligible for a Medicare number), and consenting to have survey data linked with administrative data (eg, records of health service use). The final inclusion criterion involved verification by the Australian Department of Human Services that sufficient details had been provided for data linkage to occur. Sufficient details included as many of the following as were needed for a match to be made with the administrative dataset: name, address, date of birth, Medicare number.

### Recruitment Campaign

**Figure 1:**
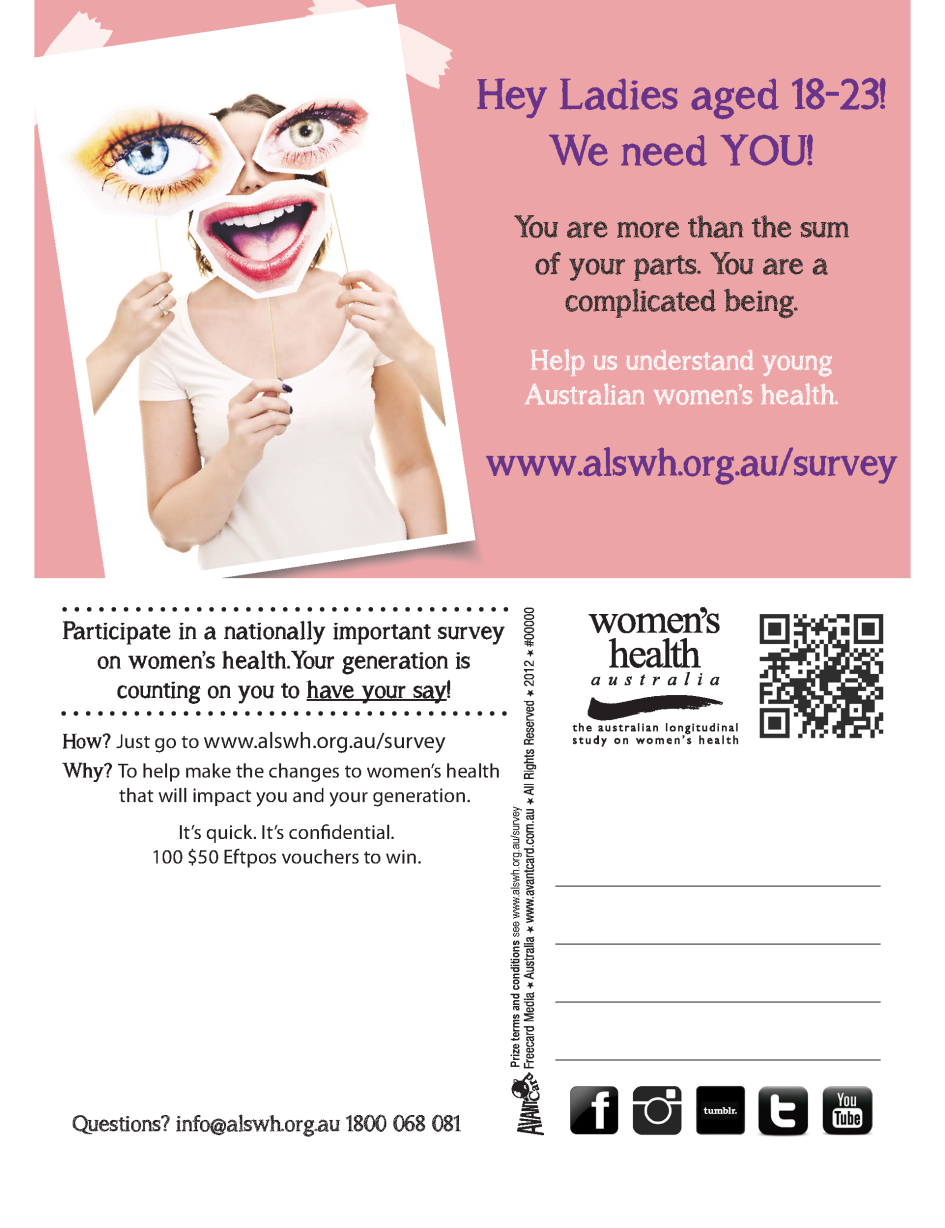
A postcard created as part of the ALSWH promotion, distributed across 1439 venues, at events, and through mailouts.

**Figure 2:**
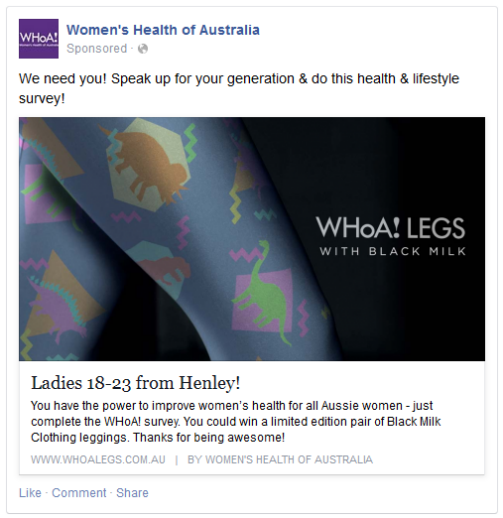
A less successful Facebook advertisement created as part of the WHoA! promotion, targeted at women aged 18-23 living within 16 kilometres of Henley Beach South Australia (0 users clicked the link).

The recruitment campaign comprised two promotions: ALSWH and Women’s Health of Australia! (WHoA!). Due to a slow rate of participation at the beginning of recruitment, it was decided that assistance from a marketing communications company could be beneficial for the second campaign. ALSWH ran for the total campaign (October 2012-December 2013) and was designed and implemented by ALSWH staff (see Figure 1 and Multimedia Appendices 1 and 2). WHoA! commenced October 2013 and was conducted by a marketing company with ALSWH staff support (see Figure 2 and Multimedia Appendices 3 and 4). Slogans, branding, and incentives differed for the two promotions. ALSWH offered the chance to win one of 100 AU $50 prepaid debit cards and WHoA! used a 1990s-themed fashion incentive of the chance to win one of 2000 exclusive items of clothing. Participants in South Australia were offered the chance to win one of 99 AU $50 prepaid debit cards instead of the fashion promotion due to the state’s lotteries legislation.

**Figure 3:**
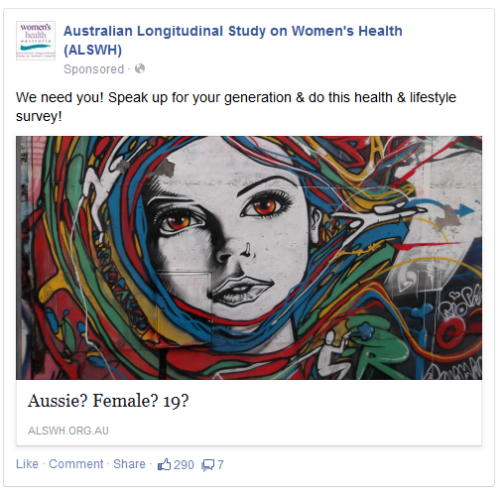
A successful Facebook advertisement created as part of the ALSWH promotion, targeted at women living in Australia aged 19 (7669 users clicked the link). Copyright User:couscouschocolat / Wikimedia Commons / CC-BY-SA-2.0.

Multiple recruitment strategies were used for both promotions and are summarized in (Table 1) (for examples of advertising and promotional materials, see Figures 1-3 and Multimedia Appendices 1-4). Strategies were categorized as Facebook, which included Facebook pages and advertising; other Web-based activities, which involved establishing accounts on other social media platforms as well as dedicated websites; referral, which utilized existing networks and snowballing; traditional media that included direct and mass marketing as well as bulk mailouts (eg, to tertiary institutions); and the WHoA! fashion company, which involved direct and indirect marketing focused on the fashion company’s involvement with ALSWH.

**Table 1:** Strategies used to recruit the ALSWH 1989-95 cohort over the recruitment period October 2012 to December 2013.^a^

Strategy	Method^b^
Facebook	Facebook page
	Facebook advertising (paid and unpaid^c^)
Other Web activities	Twitter
	Web forums
	Instagram
	Tumblr
	YouTube
	Web advertising/promotion
	ALSWH & WHoA! websites
Referral	Emails from ALSWH staff to personal/professional networks (ALSWH only)
	Emails to original ALSWH cohorts (ALSWH only)
	Emails to ALSWH collaborators and their networks (ALSWH only)
	Emails to professional bodies
	Snowballing (via participants who already completed the survey)
Traditional media	Posters
	Postcards
	Business cards
	Flyers
	Magazine
	Newspapers
	Television (ALSWH only)
	Radio (ALSWH only)
WHoA! fashion co.	Where the participant ascribed their participation to the involvement of the fashion company, eg, through emails sent on behalf of WHoA! by the fashion company to their subscribers (WHoA! only)

^a^Examples of materials and advertisements used for recruiting are available in Multimedia Appendices 1-4.

^b^Unless stated otherwise, methods were conducted under both ALSWH and WHoA! promotions.

^c^Unpaid Facebook advertising included posting information about the study on Facebook pages of other organizations.

The campaign was dynamic by design so that successful strategies could be augmented. To monitor the success of the campaign in relation to the strategies that were being used, responses to an item that asked participants how they had heard about the study were reviewed on a weekly basis. For the purposes of this paper, the response options for this item (which varied slightly between the two recruitment promotions) were allocated to one of the five recruitment strategies defined in Table 1. Demographic characteristics of the respondents were monitored on a weekly basis and compared to the Australian Census to examine demographic representativeness. So, as well as being responsive to the number of participants entering the study, it was also possible to target recruitment strategies to respond to the composition of the sample. In particular, paid Facebook advertisements targeted underrepresented groups throughout recruitment (see Figure 3 for an example of a Facebook advertisement targeting an underrepresented group). Overall, ads were targeted to augment particular ages, areas of residence, and level of education. The decision about when to target underrepresented groups was determined by there being at least a 2% difference between the sample category and equivalent category in the census.

### Procedure

In all promotional materials, potential participants were directed to a website that included a link to the survey and participant information, including the inclusion criteria, information statement, and details concerning incentives for participation. The documentation was updated once the WHoA! promotion started, but essentially participants experienced a similar process of reading documentation and then commencing the online survey.

The survey contained questions about health and well-being as well as items that concerned demographic characteristics and life experiences [23]. In addition, participants were asked to provide formal consent to their data being linked to administrative datasets and to provide compulsory contact details and their Medicare number to facilitate data linkage. Follow-up phone calls were made to participants who had provided contact details but who had not completed other survey requirements. Data were sent to the Department of Human Services to verify participants’ Medicare numbers and personal details. All methods, including obtaining informed consent as implied by survey initiation, were approved by the University of Newcastle and the University of Queensland Human Research Ethics Committees.

### Statistical Analysis

Sociodemographic characteristics (age, area of residence, education, study, and relationship status) were compared for each recruitment strategy with results for women in the same age group in the 2011 Australian Census. Multinomial logistic regression was used to compare sociodemographic characteristics for each strategy relative to the reference category (Facebook). These models simultaneously estimate the odds ratio for each characteristic for each recruitment strategy relative to an odds ratio of one for the same characteristic in the Facebook group. All analyses were conducted using SAS software, version 9.3. The significance level was set at .05.

##  Results

A total of 54,685 surveys were initiated; however, 16,753 exited before providing enough information to determine whether they belonged to valid survey respondents. Of the remaining 37,932 initiated surveys, 19,955 were considered invalid for the following reasons: 3148 excluded personal details needed to verify participants, 36 were not eligible for a Medicare number; 13,476 were excluded for issues relating to consent to data linkage (ie, did not answer consent item, refused data linkage); 3159 were excluded due to identity concerns (eg, survey completed by someone else, duplicate); and 9 were considered invalid for other reasons. Before the verification process, 127 survey respondents withdrew. Data from the remaining 17,977 potential participants were sent to the Department of Human Services to have their Medicare numbers and personal details verified. Of those, 255 failed the verification process and 155 were excluded post verification (eg, duplicate). That left a total of 17,567 verified participants, 498 of whom were allocated to the future pilot group, in keeping with ALSWH procedures of maintaining a separate group of participants for pilot testing each new survey. The final cohort was 17,069 participants.

**Figure 4:**
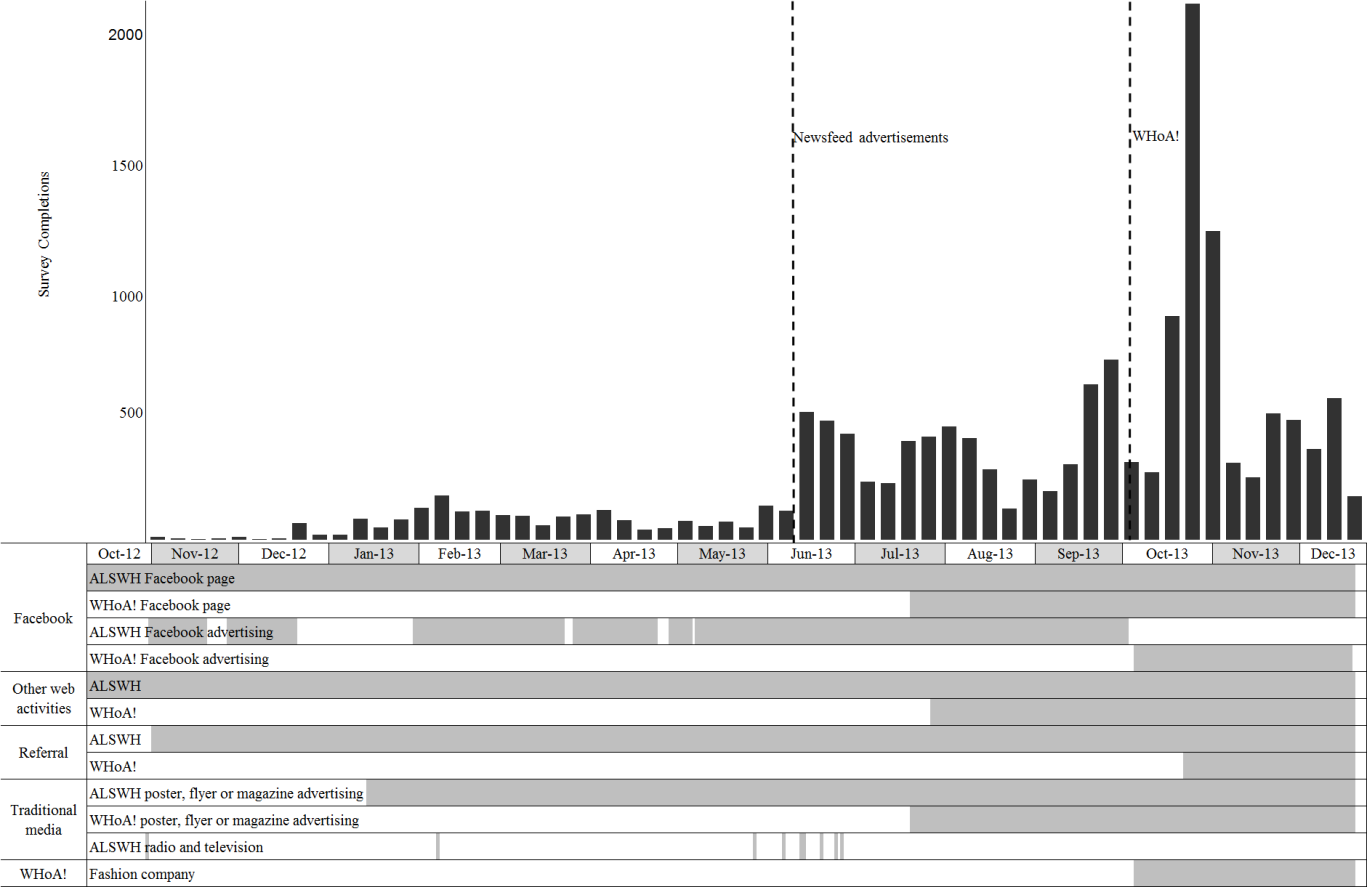
Survey completions by week in relation to recruitment strategies for the ALSWH 1989-95 cohort over the recruitment period October 2012 to December 2013.

Figure 4 shows the periods when various recruitment methods were active, along with the number of participants recruited each week. Women were exposed to Facebook pages with recruiting posts and advertisements, other online media, and ALSWH referral for the entire recruitment period. Facebook advertising was reasonably constant, with breaks due to technical issues, pricing, and the changeover from the ALSWH promotion to WHoA!. In June 2013, Facebook introduced newsfeed advertising, so that advertisements appeared in the central part of the screen (rather than to the side) and on mobile devices that used the Facebook app. Targeted Facebook advertising was also introduced in June 2013, whereby particular sections of the community were identified and advertisements were shown only to those people who met the specified criteria. For example, advertisements designed for women of a particular age (eg, 19 years) appeared only on the Facebook pages of women who were that age (see Figure 3).

The introduction of targeted newsfeed advertising in combination with increased advertising had a significant impact on participant responses. Prior to this time, an average of 10.5 women responded per day (2434 surveys over 232 days). After the introduction of targeted newsfeed advertisements and before the WHoA! promotion, responses rose to an average of 54.3 per day (6787 surveys over 125 days). The WHoA! promotion was officially launched in early October 2013, and the fashion company database was emailed late October 2013, resulting in a spike of responses. However, WHoA! prelaunch activities were conducted from July until the launch to engage the interest of potential participants in the WHoA! materials. During the WHoA! promotion, responses rose to an average of 100.6 per day (7849 surveys over 78 days). As can be seen in Figure 4, this was the most intensive period of recruiting activities during the campaign incorporating both ALSWH and WHoA! activities.

Of the 17,069 women enrolled in the 1989-95 cohort, 200 did not indicate how they heard about the survey. Of the remaining 16,869 women, 69.94% (n=11,799) indicated Facebook, 4.93% (n=831) other Web activities, 7.02% (n=1184) referral, 5.39% (n=910) traditional media, and 12.72% (n=2145) the fashion company (see Table 2). Compared with the 2011 Australian Census, respondents to each of the recruitment strategies were more likely to have a university degree and to be studying. Most of the strategies resulted in relatively representative samples of women with regards to age, except that referral and the fashion company overrepresented older women compared to the population. There was also a higher proportion of women living in urban areas among the women recruited via the fashion company. With regards to education, all recruitment strategies led to an underrepresentation of less educated women compared to the 2011 Australian Census; however, Facebook was the least biased in this regard. Facebook resulted in the most representative sample in relation to the Australian Census, whereas the fashion company sample was the least representative. As would be expected and has been reported in detail in Mishra et al [24], the overall sample was broadly representative of women in this age group with overrepresentation of women with higher levels of education.

**Table 2:** Demographic characteristics by recruitment strategy^a^of 18-23 year old women from the ALSWH 1989-95 cohort compared with 18-23 year old women from the 2011 Australian Census.

		Facebook (N=11,799)	Other Web activities (N=831)	Referral (N=1184)	Traditional media (N=910)	Fashion company (N=2145)	2011 Australian Census (N=847,042)
**Age in years, %**							
	18	15.92	18.4	13.01	15.9	10.49	15.98
	19	18.76	16.5	14.53	16.9	12.91	16.21
	20	17.35	15.0	16.30	17.1	17.25	16.82
	21	15.55	17.6	19.26	16.5	20.09	17.07
	22	16.09	17.8	20.10	16.0	19.77	16.95
	23	16.32	14.7	16.81	17.5	19.49	16.96
**Area of residence, %**							
	Major city	74.17	78.1	74.83	73.1	80.98	74.52
	Inner regional	17.50	14.6	15.46	17.1	13.10	15.95
	Outer regional	6.86	5.9	8.36	8.0	5.08	7.23
	Remote/ very remote	1.14	1.0	1.10	1.8	0.42	2.02
	Missing/ migratory/ no usual address	0.33	0.5	0.25	0	0.42	0.28
**Highest qualification achieved, %**							
	Less than Year 12	8.45	6.4	4.90	4.6	5.64	14.93
	Year 12	43.22	47.3	46.20	47.1	40.23	46.09
	Certificate/ Diploma	26.88	20.0	18.67	24.2	30.07	21.75
	University degree	21.45	26.4	30.24	24.1	24.06	9.41
	Missing	0.01	0	0	0	0	7.82
**Studying, %**							
	No	34.74	26.6	26.94	30.6	37.44	47.36
	Yes	65.16	73.4	73.06	69.3	62.42	47.44
	Missing	0.10	0	0	0.1	0.14	5.20
**Relationship status, %**							
	Never married	71.87	78.0	81.00	75.7	74.27	94.53
	De facto^b^	24.32	19.6	15.88	21.0	24.20	
	Married	3.37	2.3	3.04	2.9	1.45	4.89
	Separated/ divorced/ widowed	0.43	0.1	0.08	0.4	0.09	0.59

^a^200 missing information on what alerted them to the survey.

^b^De facto relationship is included with never married in the 2011 Australian Census.

Odds ratios for sociodemographic characteristics of women in each recruitment group relative to women recruited via Facebook are shown in Table 3. Compared to women aged 20, women aged 21-23 were more likely to be recruited via other Web activities, referral, and through the fashion company than via Facebook (significant ORs between 1.20 and 1.33). Compared to women with Year 12 qualifications, less educated women were less likely to be recruited by strategies other than Facebook (ORs between 0.50 and 0.72). Women recruited via the fashion company also differed from those recruited via Facebook in that they were less likely to live outside a major city (ORs between 0.34 and 0.69), were less likely to be studying (OR 0.89), and were less likely to have a partner (OR 0.96 for in a de facto relationship and OR 0.42 for married).

**Table 3:** Odds ratios for demographic characteristics by recruitment strategy of 18-23 year old women from the ALSWH 1989-95 cohort recruited October 2012-December 2013, using Facebook as the reference category.

		Other Web activities (N=831) OR (95% CI)	Referral (N=1184) OR (95% CI)	Traditional media (N=910) OR (95% CI)	Fashion company (N=2145) OR (95% CI)
**Age in years**					
	18	1.33 (1.04-1.70)^a^	0.87 (0.70-1.08)	1.01 (0.80-1.28)	0.66 (0.56-0.79)^a^
	19	1.01 (0.79-1.30)	0.82 (0.66-1.02)	0.91 (0.72-1.15)	0.69 (0.59-0.82)^a^
	20	reference	reference	reference	reference
	21	1.30 (1.02-1.67)^a^	1.32 (1.08-1.61)^a^	1.07 (0.85-1.35)	1.30 (1.12-1.51)^a^
	22	1.28 (0.99-1.63)	1.33 (1.09-1.62)^a^	1.01 (0.80-1.28)	1.24 (1.06-1.44)^a^
	23	1.04 (0.80-1.34)	1.10 (0.89-1.35)	1.08 (0.86-1.36)	1.20 (1.03-1.40)^a^
**Area of residence**					
	Major city	reference	reference	reference	reference
	Inner regional	0.79 (0.65-0.97)^a^	0.88 (0.74-1.03)	0.99 (0.83-1.19)	0.69 (0.60-0.78)^a^
	Outer regional	0.82 (0.60-1.10)	1.21 (0.97-1.50)	1.19 (0.92-1.53)	0.68 (0.55-0.83)^a^
	Remote/ very remote	0.80 (0.39-1.65)	0.96 (0.54-1.70)	1.57 (0.93-2.66)	0.34 (0.17-0.67)^a^
**Highest qualification achieved**					
	Less than Year 12	0.69 (0.51-0.93)^a^	0.54 (0.41-0.72)^a^	0.50 (0.36-0.69)^a^	0.72 (0.59-0.88)^a^
	Year 12	reference	reference	reference	reference
	Certificate/diploma	0.68 (0.56-0.82)^a^	0.65 (0.55-0.76)^a^	0.82 (0.70-0.98)^a^	1.20 (1.08-1.34)^a^
	University degree	1.12 (0.94-1.33)	1.32 (1.14-1.52)^a^	1.03 (0.87-1.22)	1.20 (1.07-1.36)^a^
**Studying**					
	No	reference	reference	reference	reference
	Yes	1.47 (1.26-1.72)^a^	1.44 (1.26-1.65)^a^	1.21 (1.05-1.40)^a^	0.89 (0.81-0.98)^a^
**Relationship status**					
	Never married	reference	reference	reference	reference
	De facto relationship	0.74 (0.62-0.89)^a^	0.58 (0.49-0.68)^a^	0.82 (0.69-0.97)^a^	0.96 (0.86-1.07)^a^
	Married	0.62 (0.39-0.99)^a^	0.80 (0.56-1.13)	0.80 (0.54-1.20)	0.42 (0.29-0.60)^a^
	Separated/divorced/ widowed	0.26 (0.04-1.86)	0.18 (0.02-1.26)	0.96 (0.35-2.68)	0.21 (0.05-0.86)^a^

^a^Significant odds ratios.

## Discussion

### Principal Findings

The primary aim of recruiting over 10,000 women aged 18-23 years was met, with 17,069 meeting the inclusion criteria and forming the 1989-95 ALSWH cohort. The most successful strategy used in terms of number of participants gained was Facebook, under both the ALSWH and WHoA! promotions. Direct marketing strategies were the next most successful, with the fashion company under the WHoA! promotion and the more general referral strategy together accounting for 20% of the final sample. While other Web activities and traditional media were the least successful activities, they still managed to attract 5% each of the final sample.

### Comparison With Prior Work

Facebook was the most successful in recruiting a sample of young women that represented those in the Australian population. This was most likely due to the ability to constantly monitor women’s particular characteristics in relation to the Australian Census and post Facebook advertising as necessary to target any underrepresented groups. Such a strategy most likely mitigated the age bias reported by past research, which found that Facebook attracted a lower proportion of older women (ie, aged 22-25) compared to Australian Census data [12]. There was, however, an age bias among those recruited via referral and the fashion company strategies. These strategies attracted a higher proportion (56.17% and 59.35% respectively) of women aged over 20 years than is apparent in the population (50.98%) and were more likely than Facebook to attract women aged over 20 years. The multiple strategies approach to the recruiting campaign resulted in a total sample that has been found to be representative of the population with regard to age [24].

Unfortunately, the same was not true for education. Compared with Australian Census data, all of the recruiting strategies resulted in subsamples that were overrepresentative of women with a tertiary education or who were studying. Overall, referral was the most likely and Facebook the least likely to attract women with a university degree. This is not surprising since referral largely consisted of using networks of people who worked on, participated in, or who were close to the ALSWH. It is noteworthy that Facebook achieved the highest proportion of women with less than a Year 12 education, although this was still 6% below the proportion indicated by the Australian Census as having this level of education. Education bias is a common occurrence for survey research [4,8,25], which warrants future research to utilize more refined targeting to recruit less educated individuals.

Facebook, referral, and traditional media attracted women in similar proportions by area as those that exist in the population. The most notable difference here was the tendency for the fashion company incentive to attract more women from urban areas than the other strategies. Nevertheless, the sample as a whole has been found to be representative of the population with regard to area [24].

It is difficult to compare relationship status with Australian Census data. The Australian Census records registered marital status, so that figures on the percentage of women living in de facto relationships were not available. Including women living in de facto relationships with those who had never married, the proportions are similar for all strategies. Fewer married women were recruited via the fashion company strategy, making these women the least representative of the population.

### Limitations

In generalizing the results of our recruiting techniques to other situations, a number of limitations must be considered. First, the sample was limited to women aged 18-23 years. The strategies used may be more or less effective in recruiting males or other age groups. Second, the requirement that women had to consent to linking their survey data with administrative datasets reduced the number of eligible participants and might have resulted in differential participant bias for different strategies. However, Medicare verification is also a strength of this method in ensuring participants are valid and removing the risk of multiple survey submissions from any one participant. Our previous work determined that the ALSWH 1989-95 cohort were demographically representative overall and heterogeneous with respect to health and health behavior [24]. The diversity of the sample and the wide range of variables collected, including the use of well-recognized psychometric measures, allows for valid statistical comparisons to be made. Further, when compared with women who consented to data linkage, those who did not consent were not significantly different in terms of age, area of residence, income management, education, general health, or psychological distress [26]. Last, we were unable to assess the cumulative impact of multiple approaches on the individual and relied on a single response from participants as to what attracted them to the survey. Anecdotally, participants reported having multiple contacts with various recruiting materials before joining the study. It would be a useful avenue for future research to measure not only which strategies bring participants to a research project but how many strategies are encountered by an individual before they commit to participation.

### Conclusions

Facebook was by far the most successful recruiting strategy, in line with other research results [19]. The current study found that the strengths of a Facebook recruiting approach included the reach of the advertising in terms of demographic diversity, the ability to target advertising to specific groups, and the capacity to be responsive to the sample composition as it was recruited. Weaknesses included a lack of control over how advertising is presented, variable costs that can be challenging to budget, and the inability to reach those who do not (or cannot) participate in Facebook [14,15]. Supporting Facebook advertising with a substantial social media platform assisted with recruiting and with study credibility [7,12], and the promotion of incentives via Facebook likely also increased the appeal and reach of the Facebook posts. We found that supplementing Facebook with other approaches helped to mitigate biases attributable to the use of a single method. This is in agreement with research that has recommended using social media to complement more traditional recruiting approaches [14].

The dynamic design allowed for successes to be augmented but also required almost constant contact with institutional ethics committees who needed to approve every material and individual procedure that involved recruiting. For example, every advertisement or post that mentioned recruiting had to be preapproved. In our original ethics application, we included examples of potential ads and posts in an effort to have materials readily available. However, the need to provide relevant and topical posts and ads on a frequent basis meant that this strategy was only partially successful in mitigating the need for ongoing ethics approvals. These types of delays are not in keeping with social media, where responsiveness is the key to success within the paradigm. It would be helpful for both ethics committees and researchers if clear guidelines with regard to ethical recruiting using social and other modern media were developed to prevent such labor intensive procedures.

The recruiting campaign resulted in the establishment of a national cohort of over 17,000 women aged 18-23 years. While some overrepresentation in tertiary-educated women is apparent and was unable to be fully mitigated by the multiple techniques employed, the sample is broadly representative across other demographic measures and has sufficient size and diversity to allow for subgroup comparisons [24]. In conclusion, flexibility in recruitment design, embracing new and traditional media, providing incentives, adopting a dynamic responsive approach, and monitoring the results of recruiting in terms of sample composition and number recruited led to the successful establishment of a new cohort.

## Multimedia Appendix 1

Advertising and recruitment materials from the ALSWH promotion.

## Multimedia Appendix 2

Example of a YouTube video created as part of the ALSWH promotion.

## Multimedia Appendix 3

Advertising and recruitment materials from the WHoA! promotion.

## Multimedia Appendix 4

Example of a YouTube video created as part of the WHoA! promotion.

## Abbreviations

ALSWH: Australian Longitudinal Study on Women’s Health
WHoA!: Women’s Health of Australia!
