# Supplementary material for: Online and Offline Recruitment of Young Women for a Longitudinal Health Survey: Findings From the Australian Longitudinal Study on Women’s Health 1989-95 Cohort
Source: J Med Internet Res. 2015 May 4;17(5):e109. doi: 10.2196/jmir.4261 (PMC4468605; doi:10.2196/jmir.4261)
Supplement: Supplementary file 1 [file jmir_v17i5e109_app1.pdf]

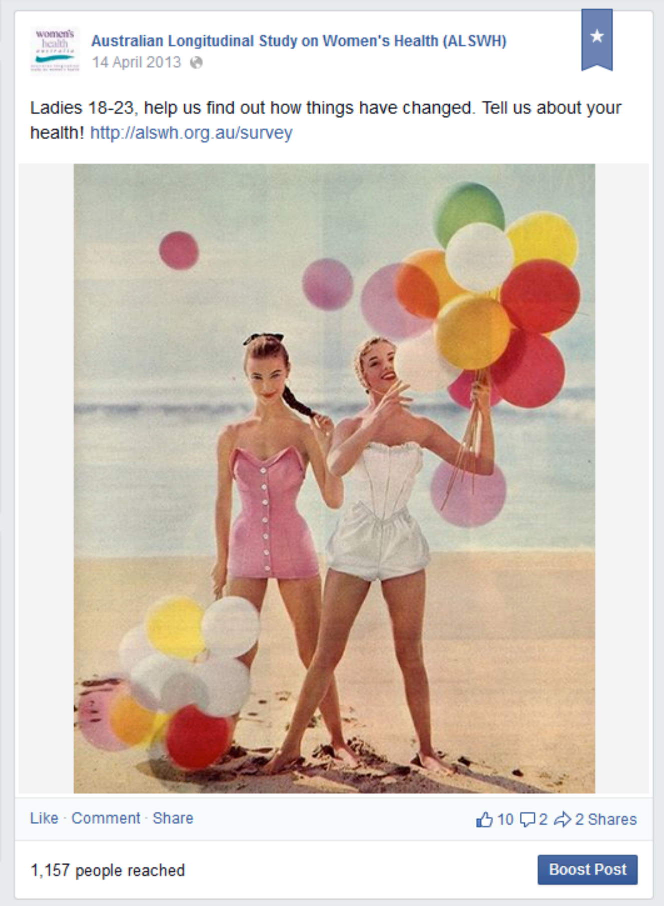

This post (dated April14, 2013) was part of the ALSWH promotion. The post was shown in the newsfeed of some users who liked the ALSWH Facebook page. All Facebook users could view the post if they visited the ALSWH Facebook page. This post reached 1,157 users and received 10 likes, two comments and two shares.
