# Supplementary material for: Online and Offline Recruitment of Young Women for a Longitudinal Health Survey: Findings From the Australian Longitudinal Study on Women’s Health 1989-95 Cohort
Source: J Med Internet Res. 2015 May 4;17(5):e109. doi: 10.2196/jmir.4261 (PMC4468605; doi:10.2196/jmir.4261)
Supplement: Supplementary file 3 [file jmir_v17i5e109_app3.pdf]

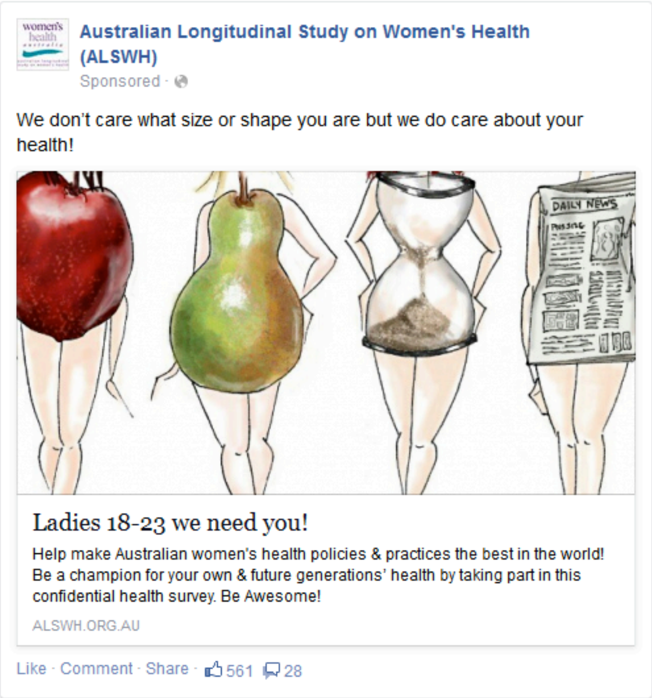

This advertisement was part of the ALSWH promotion. It was shown in the newsfeed and was targeted (by interest) to users who were living in Australia, female, aged 18-23 and interested in cooking. This advertisement was available for users who were using the Facebook application, as well as the Facebook website. The potential audience for this advertisement was 420,000 people. 244,580 users were shown the advertisement and it was presented to each person 1.56 times on average. The website link was clicked 13,937 times. This resulted in a mean click through rate of 4.332%.
